# Unimpaired Responses to Vaccination With Protein Antigen Plus Adjuvant in Mice With Kit-Independent Mast Cell Deficiency

**DOI:** 10.3389/fimmu.2018.01870

**Published:** 2018-08-28

**Authors:** Nadja Schubert, Katharina Lisenko, Christian Auerbach, Anke Weitzmann, Shanawaz Mohammed Ghouse, Lina Muhandes, Christa Haase, Tobias Häring, Livia Schulze, David Voehringer, Florian Gunzer, Werner Müller, Thorsten B. Feyerabend, Hans-Reimer Rodewald, Anne Dudeck, Axel Roers

**Affiliations:** ^1^Medical Faculty Carl Gustav Carus, Institute for Immunology, University of Technology Dresden, Dresden, Germany; ^2^Medical Faculty Carl Gustav Carus, Institute of Medical Microbiology and Hygiene, University of Technology Dresden, Dresden, Germany; ^3^Department of Infection Biology, University Hospital Erlangen and Friedrich-Alexander University Erlangen-Nuremberg (FAU), Erlangen, Germany; ^4^Faculty of Life Sciences, University of Manchester, Manchester, United Kingdom; ^5^Division of Cellular Immunology, German Cancer Research Center, Heidelberg, Germany; ^6^Medical Faculty, Institute for Molecular and Clinical Immunology, Otto von Guericke University, Magdeburg, Germany

**Keywords:** mast cells, adaptive immune responses, vaccination, adjuvant, compound 48/80

## Abstract

Innate inflammatory responses are crucial for induction and regulation of T cell and antibody responses. Mast cell (MC)-deficient *Kit* mutant mice showed impaired adaptive immunity, suggesting that MCs provide essential adjuvant activities, and pharmacological MC activation was proposed as a new adjuvant principle. However, the *Kit* mutations result in complex alterations of the immune system in addition to MC deficiency. We revisited the role of MCs in vaccination responses using *Mcpt5-Cre R26^DTA/DTA^* and *Cpa3^Cre/+^* mice that lack connective tissue MCs or all MCs, respectively, but feature an otherwise normal immune system. These animals showed no impairment of T and B cell responses to intradermal vaccination with protein antigen plus complete Freund’s adjuvant. Moreover, we demonstrate that the adjuvant effects of the MC secretagogue c48/80 in intradermal or mucosal immunization are independent of the presence of MCs. We hence find no evidence for a regulation by MCs of adaptive immune responses to protein antigens. The finding that immunological MC functions differ from those suggested by experiments in *Kit* mutants, emphasizes the importance of rigorous tests in Kit-independent MC-deficiency models.

## Introduction

Understanding the interplay of cellular and molecular factors involved in induction and regulation of adaptive immune responses is crucial to promote diverse areas of medicine including control of infectious disease, vaccine development, and immunotherapy of cancer. Dendritic cells (DCs), the most important antigen-presenting cells, represent the central switch controlling T cell, and thereby also B cell responses. DCs reside in peripheral tissues where they sample their environment. They sense microbial infection by means of pattern-recognition receptors (PRRs) which detect microbial structures [pathogen-associated molecular patterns (PAMPs)] as well as molecular changes associated with cell stress and cell death caused by the infection as a measure of how severe a threat an infection poses [danger-associated molecular patterns (DAMPs)] (1). DCs integrate PRR signals and, depending on the outcome of this process, migrate to draining LNs, upregulate their antigen-presentation machinery and increase expression of co-stimulatory molecules and cytokines, thereby providing essential signals for T cell activation and differentiation in the LN. The intensity and quality of PRR and cytokine signals the DC received in the tissue determines intensity and quality of the T cell response it drives in the LN (1).

Dendritic cells also respond to cytokines and other factors that tissue cells secrete upon detection of PAMPs or DAMPs. Mast cells (MCs) are tissue-resident hematopoietic cells that, like DCs, can express a wide array of PRRs and potentially release important immunostimulatory mediators. MCs are located in most tissues but their numbers are highest at inner and outer body surfaces suggesting a role in defense against environmental challenges. MCs were reported to be critical promoters of adaptive immunity that provide adjuvant effects through rapid provision of histamin and TNF, thereby boosting DC activation, DC migration to draining LNs, and lymphocyte homing to LNs (2–11). Moreover, MCs themselves were suggested to migrate to tissue-draining LNs and contribute to the initiation of adaptive immune responses by directly serving as antigen-presenting cells (12–18). Pharmacological MC activation was proposed as a novel adjuvant principle for vaccination (19). In keeping with these studies, important roles in diverse physiological and pathogenic immune responses have been attributed to MCs (20–25). However, these studies, which collectively built a view of the MC as an important regulator/amplifier of local innate responses that adjuvants adaptive immunity, were largely based on experiments in mutant mouse strains that are MC-deficient because of hypomorphic expression of the receptor tyrosin kinase Kit, the receptor for the growth factor stem cell factor. Since compromised Kit expression affects multiple hematopoietic and other lineages (26–29), phenotypes of *Kit* mutant strains are not necessarily the consequence of the absence of MCs. Reconstitution of *Kit* mutants with MCs differentiated from bone marrow cells *in vitro* (BMMCs) has been widely used as an argument that a phenotype was caused by MC deficiency (30). However, the many observed inconsistencies in results obtained in *Kit* mutants and BMMC reconstitutions therein, and Kit-independent MC-deficient mutants (see below) show that *Kit* mutants replete with BMMCs have been an unreliable experimental system. While *Kit* mutant strains were a common tool for the investigation of MC functions over the past decades, novel mouse models with intact Kit expression were recently developed, in which MC deficiency was achieved by different principles (31–34). *Cpa3^Cre/+^* mice (32) express high levels of Cre recombinase that eliminate MCs and a fraction of basophils through Cre-mediated genotoxicity and a p53-dependent damage response (32). These animals are completely devoid of MCs and feature a reduction of basophil numbers to 40%. In *Mcpt5-Cre R26^DTA/DTA^* mice (31, 35), expression of Cre recombinase is controlled by the mast cell protease (Mcpt) 5 promoter and thereby restricted to connective tissue mast cells (CTMCs). Cre recombinase deletes a *loxP*-flanked stop cassette of the *R26^DTA^* knock in allele (36), which results in subsequent expression of diphtheria toxin A (DTA) selectively in CTMCs, which thereby kill themselves. As a consequence, *Mcpt5-Cre R26^DTA/DTA^* mice constitutively lack CTMCs, while mucosal MCs are unaffected. In both strains, IgE-mediated systemic anaphylaxis is abrogated, consistent with lack of MCs (32, 35). However, when other MC *in vivo* functions identified earlier in the *Kit* mutant models were revisited in the new, Kit-independent strains, phenotypes of the *Kit* mutants were not reproducible in many instances (31, 32, 34, 35, 37–46). These findings suggested that reduced Kit expression in the hematopoietic system rather than MC deficiency was responsible for some of the immunological phenotypes of the *Kit* mutant strains (40), and called for a systematic reproduction of key experiments in the new, Kit-independent MC-deficiency models. The aim of this study was to revisit the role of MCs in vaccination responses.

## Results

### In *E. coli*-Infected Tissue, MCs Are Not Required for Hypertrophy of Draining Lymph Nodes

To study the role of MCs in adaptive immune responses, we used the *Mcpt5-Cre R26^DTA/DTA^* system (31, 35) to generate mice with selective deficiency for CTMCs. For maximum efficiency of MC depletion, we bred the *ROSA26-DTA* knock in allele (R26^DTA^) to homozygosity. *Mcpt5-Cre R26^DTA/DTA^* mice featured virtually complete absence of CTMCs in peritoneal cavity and skin without alteration of other hematopoietic cell subsets in peritoneum, skin, spleen, bone marrow, or blood as determined by flow cytometry and histology (Figure S1 in Supplementary Material). We had earlier shown highly efficient depletion of skin MCs on formalin-fixed, Giemsa-stained sections (47). As mucosal MCs of the intestinal tract can be metachromatically stained using special fixation procedures like Carnoy fixation but not after formalin fixation, we now performed additional Giemsa staining of Carnoy-fixed skin to exclude the presence of skin MC populations that fail to stain with conventional protocols (Figure S1 in Supplementary Material) and confirmed near complete absence of MCs in the skin of *Mcpt5-Cre R26^DTA/DTA^* mice.

**Figure 1:**
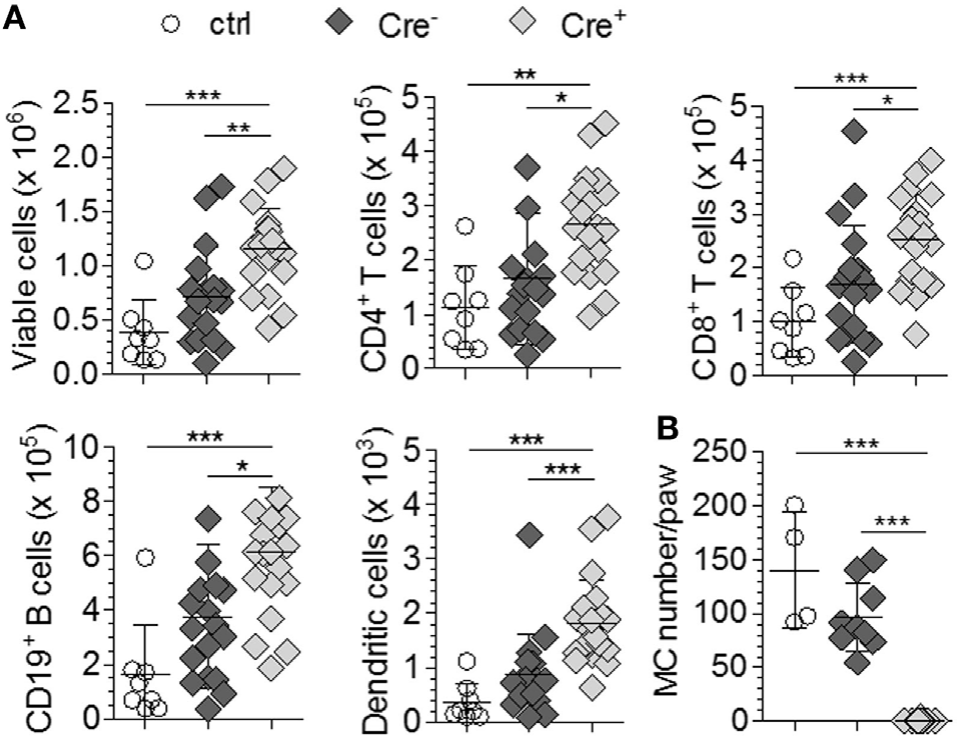
Hypertrophy of LNs draining *E. coli*-infected tissue does not depend on mast cells (MCs). **(A)** Total LN cellularity and absolute size of different cell populations of the ipsilateral popliteal LN analyzed 24 h after injection of 1 × 10^5^ *E. coli* J96 into the hind footpad of *Cre*-negative *R26^DTA/DTA^* (*n* = 9) and *Mcpt5-Cre^+^R26^DTA/DTA^* mice (*n* = 10). Four *Cre*-negative control mice were injected with saline. Data from two independent experiments with similar results. Means ± SD are shown. **(B)** Numbers of MCs in *E. coli* J96-infected or saline-injected footpad tissue of the animals in **(A)** 24 h after infection as determined by histological analysis of Giemsa-stained tissue sections. Means ± SD are shown. In all cases, statistical analysis was performed using one-way ANOVA and Bonferroni’s multiple comparison test.

We first investigated the impact of MCs on stimulation of the adaptive immune system by comparing LN hypertrophy in response to bacterial tissue infection in the presence or absence of MCs. McLachlan et al. described that injection of uropathogenic *E. coli* into the footpad of control mice resulted in a marked increase in total cellularity of the draining popliteal LN (3). LN hypertrophy was diminished in MC-deficient *Kit^W/W-v^* mice; however, the response was restored by reconstitution of the animals with wild-type BMMCs. We injected the same uropathogenic *E. coli* strain J96 into the footpad of *Mcpt5-Cre^+^R26^DTA/DTA^* and *Cre*-negative littermates. Saline injected animals served as controls. 24 h after infection, the *E. coli*-injected *Cre*-negative controls displayed a substantial increase in popliteal LN size (data not shown) and total cellularity with increased numbers of T cells, B cells, and DCs compared with the saline injected group as expected (Figure 1A). To our surprise, LN hypertrophy was not reduced in *E. coli*-injected *Mcpt5-Cre^+^R26^DTA/DTA^* animals, but in fact, total cellularity and T cell, B cell, and DC numbers were even increased compared with the *Cre*-negative controls (Figure 1A). MCs were virtually absent in the infected footpad tissue of *Mcpt5-Cre^+^R26^DTA/DTA^* mice 24 h after infection as verified by histology (Figure 1B). Collectively, these data demonstrate that the recruitment of lymphocytes and DCs to LNs draining the footpad tissue inflamed by the innate response to bacterial infection did not require the presence of MCs in the infected tissue.

### Absence of MCs Does Not Impair Expansion of Antigen-Specific T Cells in Response to Vaccination With Peptide and Adjuvant

We first tested whether absence of MCs affects T cell responses. We used the model antigen 2W1S, a variant of peptide 52–68 from the MHC II I-Eα chain, which is not expressed and therefore immunogenic in C57BL/6 mice (48). *Mcpt5-Cre^+^R26^DTA/DTA^* and control mice were injected intradermally with this peptide along with CFA at the tail base. Inguinal LN cellularity and the antigen-specific T cell response were monitored thereafter.

**Figure 2:**
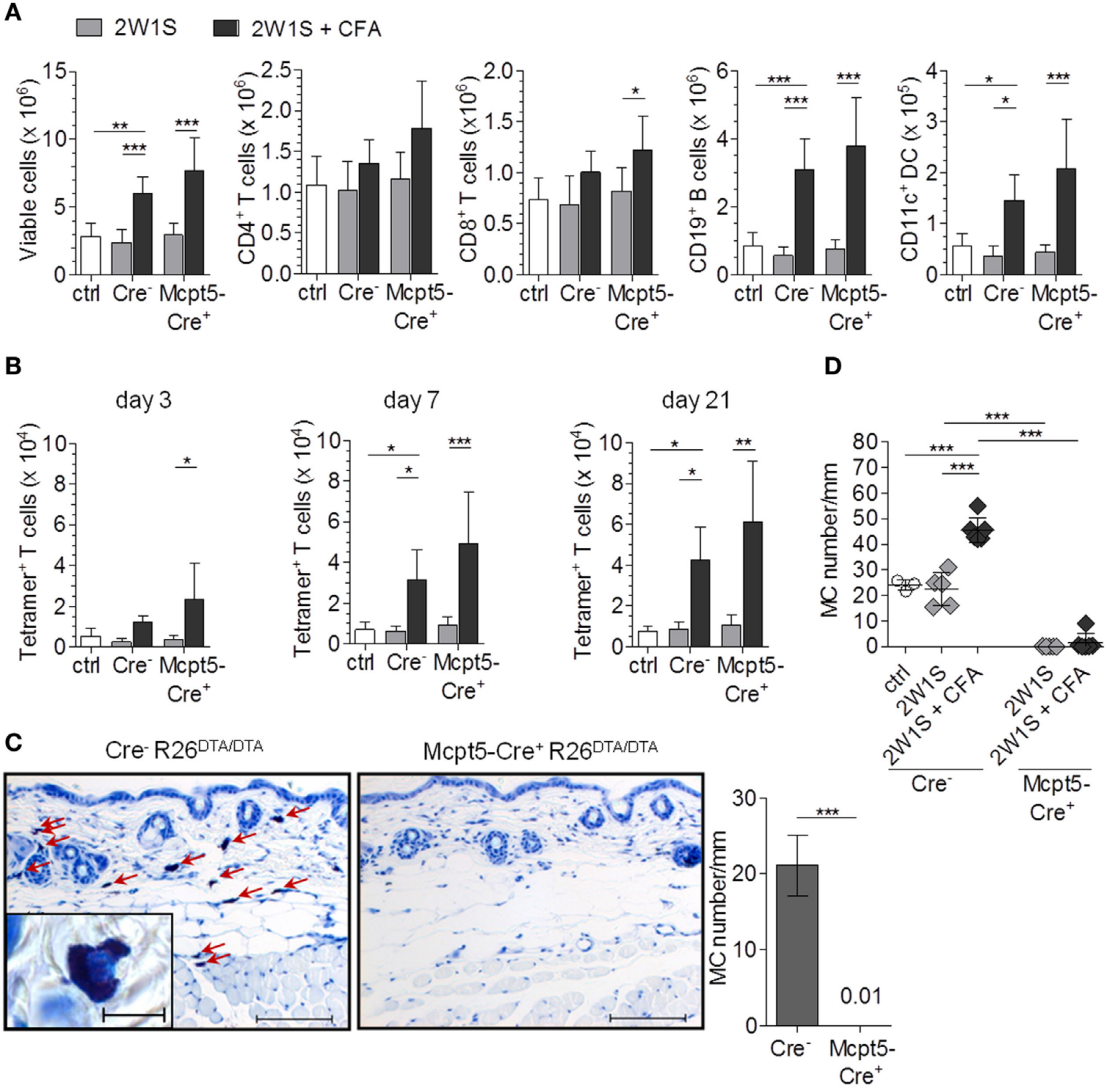
The antigen-specific T cell response is not diminished in mast cell (MC)-deficient mice. **(A)** *Mcpt5-Cre^+^R26^DTA/DTA^* mice and *Cre*-negative littermates were immunized with 2W1S peptide alone or peptide plus CFA by intradermal injection at the tail base (2W1S: *n* = 5 both groups; 2W1S/CFA: *Cre^−^ n* = 11/*Cre^+^ n* = 12) and lymph node cellularity was assessed 3 days (see Figure S2 in Supplementary Material), 7 days, and 21 days (see Figure S2 in Supplementary Material) later. Saline-injected *Cre*-negative littermates served as additional controls. **(B)** Numbers of 2W1S-specific T cells in the LNs from A [time points as in **(A)**] were quantified by MHC II tetramer staining. **(C,D)** Efficient MC depletion of the dermal immunization site at the tail base was verified by histological analysis of Giemsa-stained skin sections in untreated mice **(C)** and at day 21 after immunization **(D)** (scale bar 100 µm, inset 10 µm). Dermal MCs were counted per 20-mm length of epidermis for each mouse. *N* = 7 per group at day 0; *n* = 4 *Cre*-negative saline controls, *n* = 5 2W1S both groups; *n* = 9 *Cre^−^*/*n* = 8 *Cre^+^* 2W1S/CFA at day 21; means ± SD are shown. Data were collected from at least four independent experiments per time point. Statistical analysis was performed using one-way ANOVA and Bonferroni’s multiple comparison test **(A,B,D)** or unpaired two-sided Student’s *t*-test **(C)**.

The LNs of mice injected with CFA in addition to 2W1S peptide showed substantial increases in total cellularity compared with saline-injected controls 3, 7, and 21 days after immunization that reflected increased numbers of T cells, B cells, and DCs (Figure 2A; Figure S2A in Supplementary Material). Peptide alone had no effect on LN cellularity. As in the case of *E. coli* infection (see above, Figure 1), MC deficiency in *Mcpt5-Cre^+^R26^DTA/DTA^* mice did not impair LN hypertrophy responses to intradermal immunization with antigen plus adjuvant. On the contrary, the increase in LN cell numbers was even more pronounced in the absence of MCs.

In parallel to LN hypertrophy, numbers of 2W1S peptide-specific CD4^+^ T cells were monitored by MHC class II tetramer labeling after immunization. For verification of the specificity of this staining, we immunized WT mice with 2W1S/CFA and stained total lymph node cells with 2W1S/MHC II tetramer-APC, flow cytometrically sorted tetramer-positive and tetramer-negative CD4^+^ T cells (Figure S2B in Supplementary Material) and restimulated them with 2W1S peptide in co-cultures with total LN cells of unimmunized WT mice. IFN-γ concentrations in the culture supernatant were quantified as a measure for T cell activation. High amounts of IFN-γ were released by tetramer-positive but not by tetramer-negative cells (Figure S2C in Supplementary Material), indicating that the tetramer indeed identified 2W1S-specific T cells. Robust increases of 2W1S-specific T cell numbers were induced by immunization with 2W1S peptide plus CFA. Importantly, this increase was not diminished but even was slightly more pronounced in MC-deficient compared with MC-competent *Cre*-negative control mice at all three time points analyzed (day 3, 7, and 21 after immunization, Figure 2B).

To verify MC deficiency at the dermal immunization site of *Mcpt5-Cre^+^R26^DTA/DTA^* mice, and exclude that the inflammatory adjuvant stimulus had led to reappearance of MCs, dermal tissue was sampled from untreated mice and at day 21 after immunization and MCs were counted in tissue sections. Figures 2C,D show that MCs were depleted with high efficiency in *Mcpt5-Cre^+^R26^DTA/DTA^* animals as expected. The local inflammatory response to CFA induced an increase in MC numbers in control but not *Mcpt5-Cre^+^R26^DTA/DTA^* mice.

Collectively, these results demonstrate that MCs are not required for the expansion of antigen-specific T cells in response to immunization with peptide plus adjuvant.

### Absence of MCs Does Not Affect Antigen-Specific Humoral Immune Responses to Vaccination

**Figure 3:**
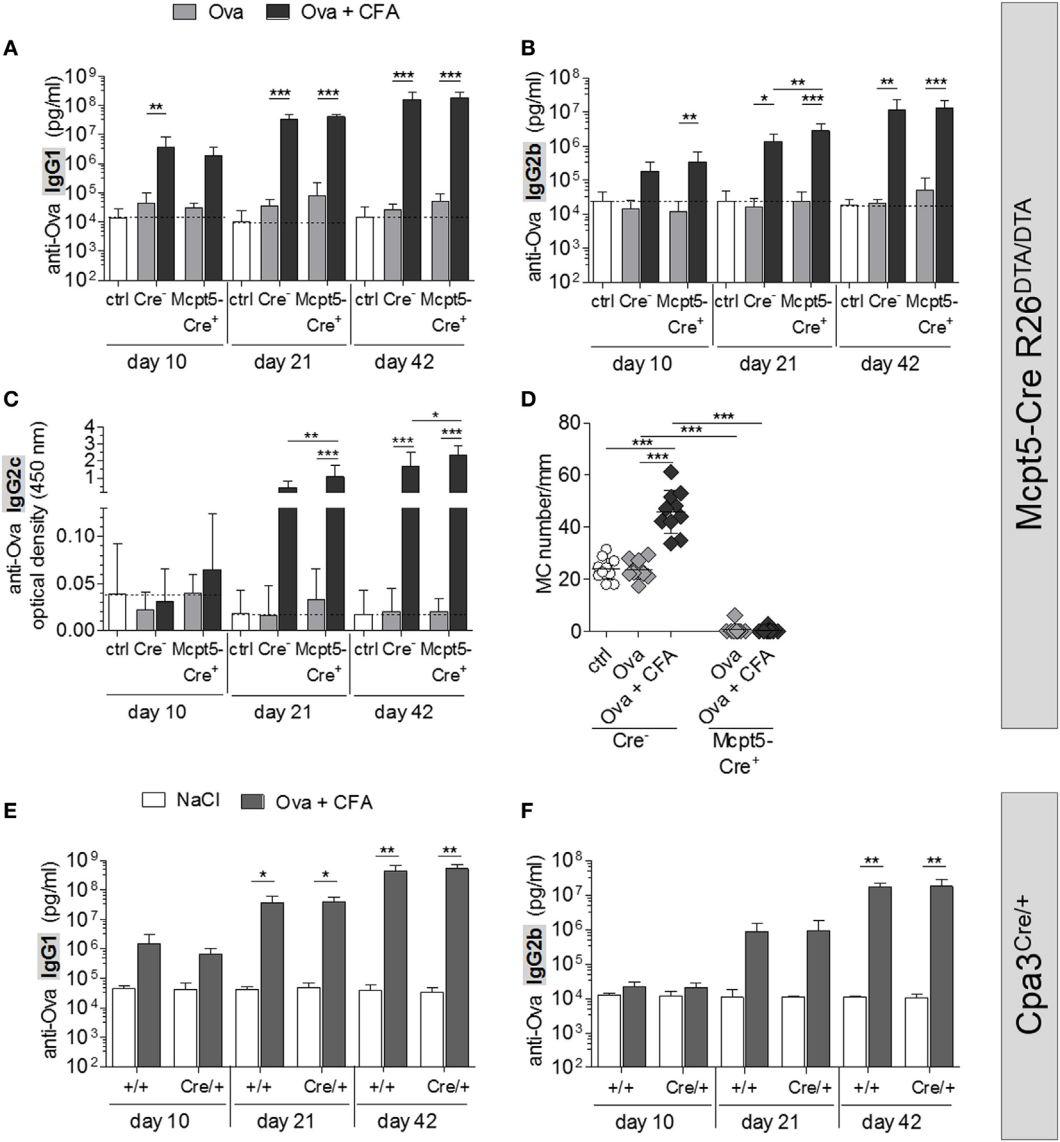
No impairment of humoral immune responses in mast cell (MC)-deficient mice. **(A–C)** Quantification of anti-Ova IgG1 **(A)**, IgG2b **(B)**, and IgG2c **(C)** by ELISA in the serum of saline treated *Cre*-controls (*n* = 12) and MC-deficient *Mcpt5-Cre^+^R26^DTA/DTA^* mice and *Cre*-negative littermates immunized intradermally with Ova alone or Ova plus CFA (Ova alone *Cre^+^ n* = 10, *Cre^−^ n* = 10; Ova plus CFA *Cre^+^ n* = 11, *Cre^−^ n* = 10). Two independent experiments were performed and yielded similar results. **(D)** MCs were counted in Giemsa-stained sections of tail base skin samples taken from each animal represented in **(A–C)** at day 42 after immunization per 20 mm length of epidermis. Means ± SD are shown. In all cases, statistical analysis was performed using one-way ANOVA and Bonferroni’s multiple comparison test. **(E,F)** Quantification of anti-Ova IgG1 **(E)** and IgG2b **(F)** by ELISA in the serum of saline treated controls (*Cpa3^+/+^ n* = 4, *Cpa3^Cre/+^ n* = 3) and MC-deficient *Cpa3^Cre/+^* mice and MC-proficient *Cpa3^+/+^* littermates immunized intradermally with Ova plus CFA (*n* = 7 both groups). A single experiment was performed.

To study antibody responses in the presence or absence of MCs, *Mcpt5-Cre^+^R26^DTA/DTA^* and *Cre*-negative control mice were immunized intradermally with chicken ovalbumin (Ova) plus CFA at the tail base and serum was collected 10, 21, and 42 days later. Serum concentrations of Ova-specific IgG of major murine subclasses (IgG1, IgG2b, and IgG2c) were determined by ELISA (Figures 3A–C). Ova alone induced no or only moderate immune responses that were not diminished in the MC-deficient animals compared with control mice. Administration of Ova plus CFA resulted in robust increases in anti-Ova IgG1 and IgG2b already 10 days after immunization, while the IgG2c response began somewhat later. By day 21, anti-Ova IgG1 and IgG2b had increased further and ranged 3–4 orders of magnitude higher, compared with mice that received Ova alone, by day 42 after immunization. The responses of MC-deficient animals were in the same range or even slightly enhanced in comparison to MC-proficient *Cre*-negative controls at all time points analyzed.

To exclude that tissue inflammation induced by CFA resulted in recruitment of MCs at the immunization site even in *Mcpt5-Cre^+^R26^DTA/DTA^ mice*, we counted MCs in Giemsa-stained sections of paraffin-embedded tail base skin, sampled from the injection site at day 42. As shown in Figure 3D, the CFA stimulus indeed resulted in a doubling of MC numbers at the immunization site in *Cre*–negative control mice, while MCs were absent or very low in number in *Mcpt5-Cre^+^R26^DTA/DTA^* mice without or with CFA administration. Using the Carnoy fixation protocol, however, we found a small population of residual MCs in LNs (but not skin, see Figure S1F in Supplementary Material) of *Mcpt5-Cre^+^R26^DTA/DTA^* mice that was not detectable with conventional formalin fixation.

To formally exclude that this small, residual population of LN MCs mediated potent immunostimulatory effects that might explain the absence of detectable immunological deficits in *Mcpt5-Cre^+^R26^DTA/DTA^* mice, we repeated immunizations in *Cpa3^Cre/+^* mice, which are completely devoid of MCs (32). Absence of MCs in skin and LNs of this strain was verified by Giemsa staining of Carnoy-fixed sections of skin tissue obtained from untreated mice and of LN tissue sampled at the end of the experiment (Figures S3A,B in Supplementary Material). *Cpa3^Cre/+^* mice and *Cpa3^+/+^* littermate controls were immunized with Ova plus CFA at the tail base. Serum was collected 10, 21, and 42 days later and concentrations of Ova-specific IgG1 and IgG2b were determined by ELISA (Figures 3E,F). Administration of Ova plus CFA to *Cpa3^Cre/+^* mice and littermate controls resulted in increases of anti-Ova IgG1 and IgG2b similar to the results obtained in *Mcpt5-Cre R26^DTA/DTA^* mice (Figures 3A,B). The intensity of this humoral immune response was not altered by the absence of MCs in *Cpa3^Cre/+^* littermates.

We also studied the antigen-specific antibody response to another antigen, protective antigen (PA) of *Bacillus anthracis* in *Mcpt5-Cre R26^DTA/wt^* mice heterozygous for the *R26^DTA^* allele. In these mice, MCs in skin tissue of the tail base 42 days after immunization were less efficiently reduced to 40% of normal numbers or lower (Figure S3D in Supplementary Material), demonstrating the impact of the zygosity of the *R26^DTA^* allele. The reduction of MCs by 60% or more did not impair the PA-specific antibody response (Figure S3C in Supplementary Material).

Mast cell-derived TNF was reported to be a critical mediator of MC effects on adaptive immunity (7). In parallel to our experiments in mice lacking MCs, we directly addressed the role of TNF secretion by MCs using mice with normal MC numbers but highly efficient, selective inactivation of the TNF gene in CTMCs (49). *Mcpt5-Cre TNF^FL/FL^* and *Cre*-negative littermate control mice were injected with Ova plus CFA intradermally at the tail base and serum was collected at different time points. Figure S3E in Supplementary Material shows that Ova-specific IgG responses were not compromised in mice lacking TNF selectively in MCs compared with *Cre*-negative control mice at the 10 and 21 day time points. A slight (albeit statistically significant) reduction of specific IgG1 but not IgG2b was observed in the mutants compared to controls.

Collectively, we show that absence of MCs does not result in relevant impairment of antibody responses to immunization with protein antigen plus adjuvant.

### The Adjuvant Effects of MC-Activating Compound 48/80 Are Independent of MCs

Compound 48/80 (c48/80) triggers MC degranulation and was shown to induce hypertrophy of draining LNs upon injection into tissues and to potently enhance adaptive immune responses against protein antigens. c48/80 was also described to be an efficient, non-toxic adjuvant for administration along with immunizing antigen *via* mucosal surfaces (19, 50, 51). The adjuvant effects of c48/80 were reduced in MC-deficient *Kit^W/W-v^* mice and reported to be dependent on MC-derived TNF (3, 7). Therefore, activation of TNF release from MCs by compounds that directly induce MC degranulation was proposed as a new principle of adjuvanting adaptive immune responses (19).

**Figure 4:**
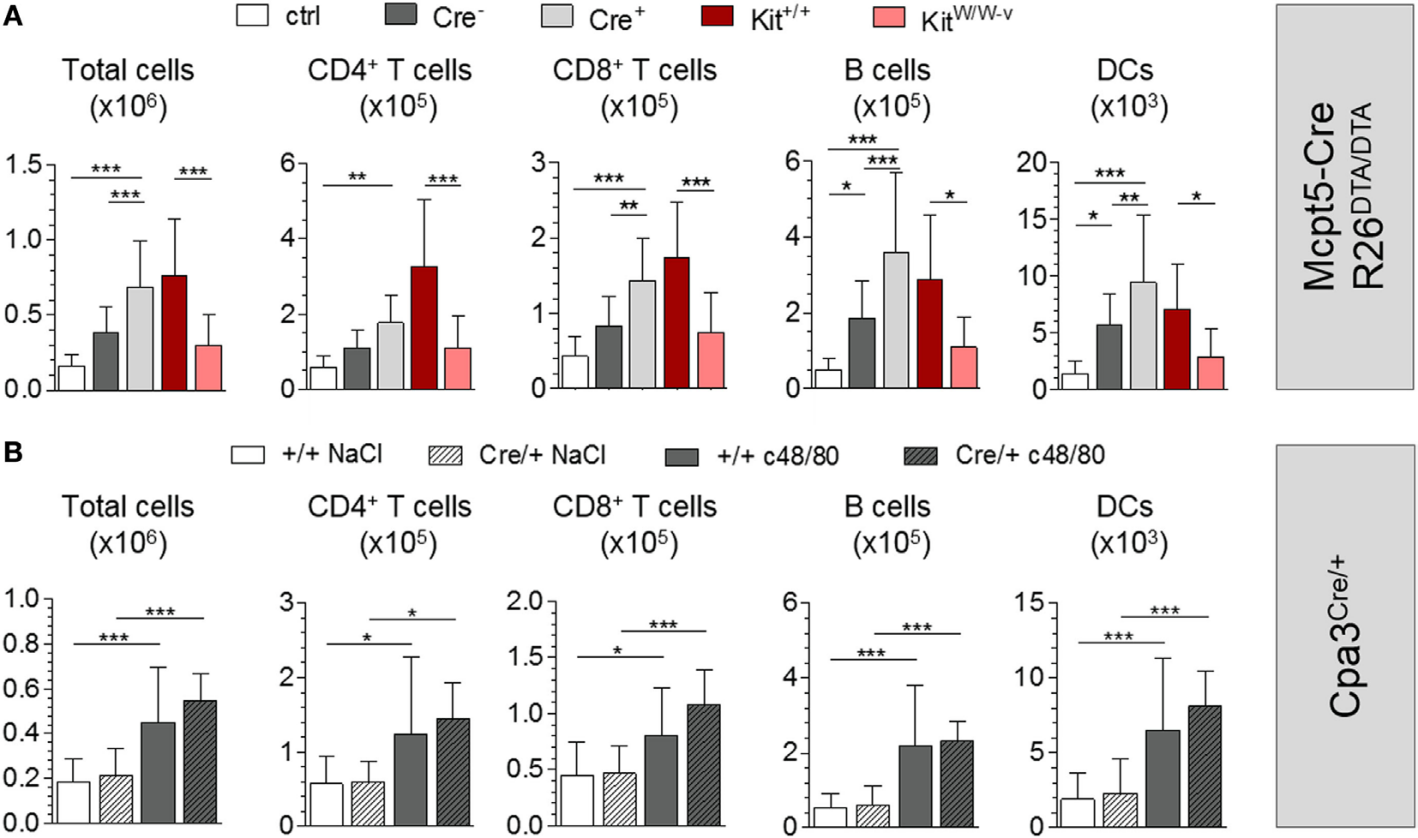
Mast cells (MCs) are not required for c48/80-mediated LN hypertrophy. **(A)** Cellularity of popliteal LNs 24 h after injection of c48/80 into footpads of *Mcpt5-Cre^+^R26^DTA/DTA^* and *Cre*-negative littermate control mice (*n* = 11 both groups) as well as *Kit^W/W–v^* (*n* = 5) and congenic *Kit^+/+^* control animals (*n* = 7) as determined by flow cytometry. Saline injected *Cre*-negative mice (*n* = 6) served as controls. Data from two independent experiments with similar results. **(B)** Cellularity of popliteal LNs 24 h after injection of c48/80 into footpads of *Cpa3^Cre/+^* and *Cpa3^+/+^* littermate control mice (*Cpa3^+/+^ n* = 16, *Cpa3^Cre/+^ n* = 11) as determined by flow cytometry. Saline was injected as control into one of both paws. A single experiment was performed. In all cases, means ± SD are shown and statistical analysis was performed using one-way ANOVA and Bonferroni’s multiple comparison test.

To investigate the adjuvant effects of c48/80 in our MC-deficiency model, we first injected c48/80 alone into the footpads of *Mcpt5-Cre^+^R26^DTA/DTA^* and *Cre*-negative littermate control mice and analyzed the draining popliteal LNs 24 h later. c48/80 induced significant LN hypertrophy in *Cre*-negative mice compared with saline-injected controls with increases in numbers of T cells, B cells, and DCs. Of note, LN hypertrophy was not reduced in MC-deficient *Mcpt5–Cre^+^R26^DTA/DTA^* animals but, on the contrary, was even enhanced compared with the responses of their MC-proficient littermates (Figure 4A). Highly efficient MC depletion in the injected tissue was verified histologically (Figure S4A in Supplementary Material). We next tested the effect of c48/80 in *Kit^W/W-v^* and congenic littermate control *Kit^+/+^* mice and reproduced the results of published studies (19) that described impaired c48/80-induced LN hypertrophy in *Kit^W/W-v^* mice (Figure 4A).

To exclude that residual LN MCs were responsible for the undiminished LN responses of *Mcpt5-Cre^+^R26^DTA/DTA^* mice, we repeated the c48/80 footpad injections in *Cpa3^Cre/+^* mice and *Cpa3^+/+^* littermate controls. Compared with the contralateral LNs draining the saline-injected footpad c48/80 induced significant LN hypertrophy including increased T cell, B cell, and DC numbers (Figure 4B). Confirming the results obtained in MC-deficient *Mcpt5-Cre^+^R26^DTA/DTA^* animals, LN hypertrophy was not reduced in *Cpa3^Cre/+^* mice but was even slightly enhanced compared to MC-proficient *Cpa3^+/+^* mice.

**Figure 5:**
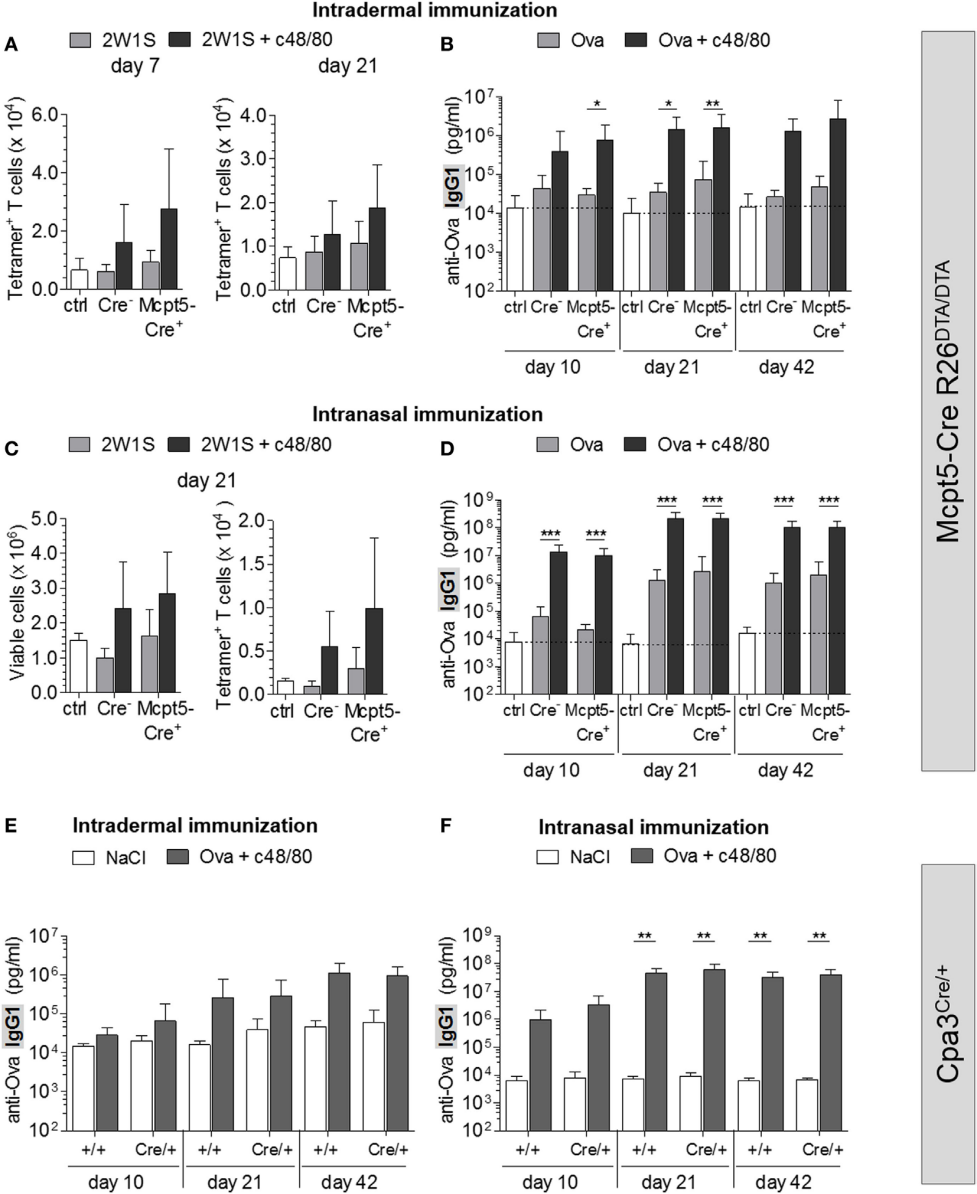
Mast cells (MCs) are dispensable for c48/80-induced antigen-specific T cell and antibody responses. **(A,B)** *Cre*-negative and *Mcpt5-Cre^+^R26^DTA/DTA^* mice were intradermally injected with antigen (2W1S or Ova) alone, with antigen plus c48/80 or with saline. **(A)** Total numbers of 2W1S-specific CD4^+^ T cells identified by tetramer staining in inguinal LNs 7 (saline *n* = 7; 2W1S alone *n* = 5 both groups; 2W1S plus c48/80 *Cre^+^ n* = 11, *Cre^−^ n* = 10) or 21 days after immunization (saline *n* = 4; 2W1S alone *n* = 5 both groups; 2W1S plus c48/80 *n* = 7 both groups). **(B)** Quantification of anti-Ova IgG1 by ELISA in the serum of saline treated *Cre*-negative controls (*n* = 12) and mice immunized i.d. with Ova alone or with Ova plus c48/80 (Ova alone *n* = 10 both groups; Ova plus c48/80 *Cre^+^ n* = 9, *Cre^−^ n* = 10). Data from two independent experiments with similar results. **(C,D)** Antigen alone (2W1S or Ova) or antigen plus c48/80 was administered into both nostrils of *Cre*-negative and *Mcpt5-Cre^+^R26^DTA/DTA^* mice. **(C)** Total cellularity and 2W1S-specific (tetramer^+^) CD4^+^ T cells were quantified flow cytometrically in draining mandibular LNs 21 days after immunization (saline *n* = 5; 2W1S alone *Cre^+^ n* = 6, *Cre^−^ n* = 5; 2W1S plus c48/80 *n* = 6 both groups). **(D)** Quantification of anti-Ova IgG1 by ELISA in the serum of saline treated *Cre*-negative controls (*n* = 11) and mice immunized intranasally with Ova alone or with Ova plus c48/80 (Ova alone *Cre^+^ n* = 13, *Cre^−^ n* = 12; Ova plus c48/80 *n* = 13 both groups). Data from three independent experiments that yielded similar results. **(E)** Quantification of anti-Ova IgG1 by ELISA in the serum of saline-treated control animals (*Cpa3^+/+^ n* = 4, *Cpa3^Cre/+^ n* = 3) and MC-deficient *Cpa3^Cre/+^* mice and *Cpa3^+/+^* littermates immunized intradermally with Ova plus c48/80 (*Cpa3^+/+^ n* = 7, *Cpa3^Cre/+^ n* = 6). A single experiment was performed. **(F)** Quantification of anti-Ova IgG1 by ELISA in the serum of saline treated controls (*Cpa3^+/+^ n* = 6, *Cpa3^Cre/+^ n* = 3) and mice immunized intranasally with Ova plus c48/80 (*Cpa3^+/+^ n* = 9, *Cpa3^Cre/+^ n* = 6). A single experiment was performed. In all cases, means ± SD are shown, and statistical analysis was performed using one-way ANOVA and Bonferroni’s multiple comparison test.

To investigate the impact of MC deficiency on c48/80-induced adaptive immune responses, we immunized *Mcpt5-Cre R26^DTA/DTA^* mice with antigen (2W1S peptide or Ova) alone or with antigen plus c48/80 intradermally at the tail base and analyzed the antigen-specific T cell and antibody responses. Numbers of 2W1S-specific CD4^+^ T cells identified by tetramer staining were increased in control animals that had received 2W1S peptide plus c48/80 compared to mice immunized with peptide alone (Figure 5A). This finding clearly demonstrates that c48/80 has potent adjuvant activity. However, this effect of c48/80 is independent of MCs since no reduction of this activity was observed in MC-deficient *Mcpt5-Cre^+^R26^DTA/DTA^* mice (Figure 5A). Immunization with Ova alone only modestly induced anti-Ova IgG1, while Ova-specific antibody concentrations increased by 2 orders of magnitude 42 days after injection of Ova plus c48/80 (Figure 5B), indicating robust adjuvant effects. MC deficiency in *Mcpt5-Cre^+^R26^DTA/DTA^* mice, however, did not compromise c48/80 adjuvant activity (Figure 5B). MC numbers were determined for each *Mcpt5-Cre^+^R26^DTA/DTA^* animal at the end of these experiments (Figure S4B in Supplementary Material).

C48/80 has been described as a potent non-toxic adjuvant for immunization *via* the nasal mucosa, suggesting that the numerous connective tissue MCs surrounding the nasal-associated lymphatic tissue (NALT) can be activated by c48/80 to stimulate adaptive immunity (19). Intranasally administrated c48/80 was shown to induce degranulation of these CTMCs in the vicinity of the NALT (19). We verified by histology that this MC population was absent in the nasal mucosa of *Mcpt5-Cre^+^R26^DTA/DTA^* mice (Figure S4C in Supplementary Material). Efficient depletion of CTMCs in *Mcpt5-Cre^+^R26^DTA/DTA^* nasal tissue was further underpinned by quantification of mRNA of the CTMC-specific endogenous *Mcpt5* gene, which we found undetectable or strongly reduced compared with control tissue (Figure S4E in Supplementary Material).

We applied antigen (2W1S or Ova) alone or along with c48/80 into the nostrils of MC-deficient *Mcpt5-Cre^+^R26^DTA/DTA^* mice and *Cre*-negative littermate control mice and analyzed the draining mandibular LNs by flow cytometry. While 2W1S peptide alone had no effects, mice that received 2W1S plus c48/80 featured increased numbers of total lymphocytes and DCs as well as 2W1S-specific (tetramer^+^) T cells in these LNs compared to saline-injected controls (Figure 5C). Likewise, administration of Ova along with c48/80 increased Ova-specific IgG1 about 100-fold compared with levels of mice that received Ova alone (Figure 5D). These findings further confirm the adjuvant qualities of c48/80. However, the stimulation of antigen-specific T and B cell responses by c48/80 was independent of the presence or absence of MCs (Figures 5C,D).

Administration of Ova without adjuvant *via* the nasal route resulted in modest IgG1 responses. Under such conditions of low intensity of danger signals, one might expect that MCs might be critical providers of adjuvant effects. However, also the antibody response to Ova alone was not reduced in MC-deficient versus control mice.

We made sure that MC deficiency of the nasal tissue persisted for the duration of the experiment (Figure S4D in Supplementary Material) and also excluded that the nasal mucosa contained mucosal type MCs (expressing Mcpt1) that in principle are not amenable to depletion in *Mcpt5–Cre^+^R26^DTA/DTA^* animals and might contribute to c48/80 adjuvant effects (Figure S4E in Supplementary Material).

To rule out effects of residual LN MCs in *Mcpt5-Cre^+^R26^DTA/DTA^*, we repeated intradermal and intranasal immunizations with Ova plus c48/80 in *Cpa3^Cre/+^* and *Cpa3^+/+^* littermate control mice. We showed that nasal tissue of *Cpa3^Cre/+^* mice was devoid of MCs, by demonstrating the absence of Mcpt1 and Mcpt5 transcripts (Figure S4E in Supplementary Material). As in *Mcpt5-Cre R26^DTA/DTA^* mice, Ova plus c48/80 induced an increase in anti-Ova IgG1 in both groups. These responses were not diminished in MC-deficient *Cpa3^Cre/+^* mice (Figures 5E,F).

Collectively, we demonstrate that presence or absence of MCs has no impact on the adjuvant effects of c48/80, which must therefore mediate these effects *via* other cell types.

## Discussion

The past two decades brought the exciting insight that adaptive immunity critically depends on innate immune responses of the tissues, which has tremendous impact on vaccine design and development of novel adjuvants (52, 53). Innate tissue responses determine, whether or not T and B cell responses are mounted and also regulate the effector class of the T and antibody response (1). The information on the tissue response is carried to the draining LN by DCs. Most tissue cells can contribute to the innate response in an infected tissue. MCs express several PRRs and are capable of releasing proinflammatory mediators (54, 55). Several of these, including the potently immunostimulatory cytokine TNF, are stored in the MCs’ secretory granules and can be rapidly released upon activation of the MC (55). TNF-containing MC particles were shown to reach draining LNs (2). The concept of the MC as an amplifier of innate immunity that provides adjuvant activity for adaptive responses was critically based on findings in *Kit*-mutant MC-deficient mice. LNs of *Kit^W/W-v^* mice draining sites of *E. coli* infection showed reduced hypertrophy responses compared to LNs of wild-type mice (3). This phenotype was reversed by reconstitution of the infection site with *in vitro* differentiated wild-type, but not TNF-deficient MCs, which has been interpreted to suggest that MCs promote recruitment and retention of lymphocytes in LNs draining infected tissues by release of TNF (3). Impaired antigen-specific humoral responses were observed in *Kit^W-sh/W-sh^* and *Kit^W/W-v^* mice (7) and pharmacologic MC activation by MC secretagogues, including c48/80, was proposed as a new adjuvant principle in vaccination (19, 56).

However, using *Mcpt5-Cre R26^DTA/DTA^* mice that are profoundly deficient for connective tissue MCs but otherwise bear a normal immune system, we found no evidence for a MC function in adaptive immune responses. Compared with wild-type controls, the LN hypertrophy response to footpad infection with *E. coli* was unchanged in the MC-deficient animals, as were expansion of antigen-specific T cells and antibody responses upon vaccination with protein antigen plus CFA. The undiminished B cell response also indicated that T helper effector function was not compromised in the absence of MCs. We confirmed unimpaired humoral responses in the *Cpa3^Cre/+^* mouse model of MC deficiency in which complete absence of all types of MCs was extensively documented (32). These mice also lack the small population of residual MCs that we detected in LNs of *Mcpt5-Cre+R26^DTA/DTA^* mice. Regarding compound c48/80, a well known stimulator of MC degranulation, we confirmed its adjuvant activity, but found that this adjuvant effect was not mediated by activation of MCs. C48/80 induced rapid LN hypertrophy that was unaffected by MC deficiency in *Mcpt5-Cre+R26^DTA/DTA^* or in *Cpa3^Cre/+^* mice. Adaptive responses to intradermal or intranasal immunization with antigen were clearly enhanced by coadministration of c48/80 in MC-competent control mice. These responses were not reduced by the deficiency for MCs in *Mcpt5-Cre+R26^DTA/DTA^* or in *Cpa3^Cre/+^* mice. Our results demonstrate that the adjuvant activity of c48/80 must be mediated by activating effects on cell types other than MCs. Compound 48/80 was shown to act on the Mrgprb2 receptor (57), which was reported to be expressed selectively in MCs (58). However, c48/80 impacts additional pathways also in non-MCs (59, 60) and the full spectrum of c48/80 effects outside the MC lineage is probably not fully understood. Our findings of undiminished adaptive responses despite MC deficiency are in line with the report by Feyerabend et al. of normal humoral responses, including immunoglobulin class switching and hypermutation, of *Cpa3^Cre/+^* mice to i.p. immunization with antigen plus alum (32).

Our study adds to a long list of cases in which results in *Kit* mutant mouse strains did not reproduce in one of the novel mouse strains with Kit-independent selective MC deficiency but otherwise normal immune system, despite reported reversion of phenotypes of *Kit* mutants by reconstitution with *in vitro* differentiated MCs (31, 32, 34, 35, 37, 39, 40, 42, 44–46) [reviewed in Ref. (38, 41, 43)]. Collectively, our results strongly suggest that *Kit* mutants are unreliable models of MC deficiency, even when combined with MC reconstitutions. MC reconstitution with poorly defined *in vitro* differentiated cells, transferred into recipients that feature a broadly abnormal immune system and empty MC niches results in a complex situation that may be the cause of misleading data. While we formally cannot exclude a contribution of differences in genetic backgrounds to the discrepancies between our results in *Mcpt5-Cre R26^DTA/DTA^* and published studies using *Kit*-mutant strains, background differences were clearly ruled out as an explanation of conflicting results obtained in *Kit* mutants versus the Kit-independent MC-deficiency model *Cpa3^Cre/+^* in another study (40). Moreover, adaptive immune responses are most commonly studied in C57BL/6 mice, which were in our case MC deficient.

The question of a MC contribution to adaptive immune responses has been addressed previously also in the context of autoimmunity. Experimental autoimmune encephalomyelitis (EAE) is a mouse model for multiple sclerosis in which CNS inflammation is induced by immunization of mice with brain-specific proteins plus CFA. While reduced EAE severity was observed in *Kit^W/W-v^* mice (61, 62) and enhanced EAE was found in the *Kit^W-sh/W-sh^* mice (63, 64), no alteration of EAE was detected in either of these strains in another study (65). Kit-independent MC deficiency in *Cpa3^Cre/+^* mice had no effect on EAE (32). In a model of antibody-induced arthritis, *Kit^W-sh/W-sh^* mice develop full disease, whereas *Kit^W/W-v^* animals are resistant to experimental joint inflammation (66, 67), demonstrating that perturbations of the immune system due to hypomorphic Kit expression beyond their MC deficiency hamper interpretations of findings in *Kit* mutant mice. Kit-independent mouse models of MC cell deficiency did not reveal any role for MCs in this arthritis model (32, 68, 69). In collagen-induced arthritis (CIA), in which a breakdown of self-tolerance is induced by injection of collagen along with CFA into mice (70), a pathogenic contribution of MCs is conceivable on the level of induction of the adaptive anti-collagen response as well as on the level of the final organ damage. MCs functions in CIA were addressed in the *Kit^W-sh/W-sh^* strain, which was found fully susceptible (71), while mice deficient for Mcpt 4 featured reduced arthritis scores (72). Based on experiments in the Kit-independent, inducible *Mcpt5-Cre R26^iDTR/iDTR^* MC-deficiency model on the moderately CIA susceptible background C57BL/6, Schubert et al. reported a role for MCs in CIA (68) and observed diminished anti-collagen T cell responses in the absence of MCs. A recent study reported a reduction of CIA in (C57BL/6 × DBA/1) F1 hybrid mice with inducible MC and basophil deficiency dependent on the time point of MC depletion (73). More recently, we backcrossed the *Mcpt5-Cre*-based MC-deficiency models to the CIA susceptible background DBA/1 for five generations and found no significant reduction of joint inflammation in these animals as yet (Figure S5 in Supplementary Material). Thus, more experiments are required to definitively settle the question of MC contributions to arthritis in this model.

Our results do not exclude that MCs play important roles in the induction of adaptive responses in contexts that remain to be identified. A special case of adaptive responses in which MC contributions play a role are pathogenic T cell responses to contact allergens. Such contact sensitizers are small chemicals that rapidly react with proteins and thereby render them immunogenic, a process called haptenization. The T cell response against the haptenized proteins depends on a rapid activation of an innate inflammatory response that these sensitizing chemicals induce (74). How contact allergens trigger this initial innate response remains unknown, but activation of MCs seems to play a role (31, 34, 49).

In line with a concept of MC- or basophil-mediated immune regulation, targeted inactivation of histamine receptors was shown to result in altered T cell polarization upon immunization with OVA and adjuvant (75). Moreover, effects of IgE-mediated MC activation on adaptive immunity are well documented (11). However, MC contributions to adaptive responses are much more restricted than previously concluded from experiments in *Kit*-mutant strains. MCs play no role in “standard” immune responses in situations with abundant danger signals for DC activation, but might serve to fill special functional gaps in the repertoire of mammalian innate responses. They might play a role for adaptive immunity under particular conditions of limiting danger signal intensities. The studies performed in Kit-independent MC-deficiency models indicate that potential functions of MCs in regulation of adaptive immunity, beyond pathophysiological functions in IgE-mediated allergic disease and delayed type hypersensitivity responses to exogenous chemicals, remain to be demonstrated.

## Materials and Methods

### Mice

All mice were housed at the Experimental Center, Medical Faculty Carl Gustav Carus, TU Dresden, under specific pathogen-free conditions. 8- to 21-week-old mice were used. Littermates were used as controls. All procedures were in accordance with institutional guidelines on animal welfare and were approved by the Landesdirektion Dresden (DD24-5131/207/2 and DD24-5131/207/33).

### Immunizations and *E. coli* Infections

Ovalbumin (Catalog No. A2512), c48/80, and CFA (containing 1 mg/ml *Mycobacterium tuberculosis*) were obtained from Sigma-Aldrich. 2W1S peptide (sequence: EAWGALANWAVDSA) was kindly provided by Dr. J. B. McLachlan, New Orleans. Anthrax PA was obtained from List Biological Laboratories. All reagents were finally dissolved in sterile 0.9% saline. For intradermal immunization, each mouse was injected with 50 µl of a 10 ng/µl solution of the respective antigen (OVA, PA, or 2W1S peptide) with or without 0.6 µg/µl c48/80. Half of these 50 µl were injected intradermally at each side of the tail base. When CFA was used as an adjuvant, 0.5 µg antigen (OVA, PA, or 2W1S peptide) were dissolved in 25 µl saline and emulsified with 25 µl CFA/mouse (1:1 emulsion). Half of these 50 µl were injected at each side of the tail base. For mucosal immunization, mice were anesthetized with isoflurane and 10 µg of antigen or antigen plus 30 µg c48/80 were administered nasally at day 0, 7, and 14 in a total volume of 15 µl/mouse (7.5 µl into each nostril). For analysis of LN hypertrophy upon injection of c48/80, 2.4 µg/g body weight of c48/80 were dissolved in a volume of 40 µl. Half of this volume was injected subcutaneously into each hind footpad of one mouse. As negative controls, additional mice were injected with 20 µl of saline into each hind footpad. For analysis of LN hypertrophy in Cpa3^Cre/+^ mice, one hind footpad was injected with c48/80, the other with saline (20 µl each). Uropathogenic *E. coli* J96 (ATCC 700336) were purchased from LGC Standards (Wesel, Germany). From an overnight culture in Luria–Bertani (LB) broth, bacteria were grown again in LB broth to an A_600_ of 0.6 where they reached the exponential phase. Bacterial cultures yielded about 1 × 10^7^ viable cells/ml. *E. coli* were washed twice and dissolved in sterile saline. 1 × 10^5^ *E. coli* in a volume of 20 µl were injected into each hind footpad of one mouse. As negative controls, additional mice were injected with 20 µl of saline into each hind footpad.

### Quantification of LN Hypertrophy and 2W1S-Specific T Cells

Inguinal LNs were isolated at day 3, 7, or 21 after immunization with 2W1S plus adjuvant and single cell suspensions were generated by crushing between glass slides. APC-coupled 2W1S-MHC class II tetramer was kindly provided by Dr. J. B. McLachlan, Department of Microbiology and Immunology, Tulane University School of Medicine, New Orleans. Isolated cells were resuspended in 2% BSA/PBS and incubated with 2W1S-MHC II tetramer-APC in a final concentration of 10 nM for 1 h at room temperature in the dark. Cells were washed, stained with monoclonal antibodies in a volume of 50 µl for 30 min at 4°C and washed twice. Numbers of total cells, various cell types, and antigen-specific CD4^+^ T cells were determined using a Miltenyi MACSQuant^®^ flow cytometer. After footpad injections, draining popliteal LNs were isolated 24 h later for flow cytometric analysis.

### Anti-Ovalbumin-Specific ELISA

96-well plates (Nunc MaxiSorp™, VWR) were coated with Ova (2 ng/µl in 100 mM NaHCO_3_, 33.6 mM Na_2_CO_3_, pH 9.5) at 4°C over night. Plates were washed thrice with 0.05% Tween 20/PBS and blocked with 200 µl 2% skimmed milk powder/PBS for 1 h at 37°C. Plates were washed thrice. Standards of anti-Ova IgG1 and IgG2b (Thermo Scientific Fisher) ranging between 62,500 and 122 pg/ml were generated by serial twofold dilutions. 100 µl/well of standards and serum samples were pipetted into the Ova-coated plates in doublets and incubated 1 h at 37°C. Plates were washed thrice. Secondary antibodies (HRP-labeled goat anti-mouse IgG1, IgG2b, and IgG2c, Dianova) were diluted 1:10,000 (anti-IgG1 and -IgG2b) or 1:2,000 (anti-IgG2c) and 100 µl/well were incubated 1 h at 37°C. After washing thrice, 100 µl freshly mixed substrate (BD OptEIA™) per well were added and plates were incubated for 15–30 min at RT in the dark. Reactions were stopped by adding 50 µl 2N H_2_SO_4_/well. Optical density was measured at 450 nm (reference value 570 nm) using a Sunrise™ spectral photometer. Magellan™ software was used for non-linear regression analysis.

### Histology

Tail base skin was fixed in 4% formalin for 1 week and embedded in paraffin. Heads of mice were freed from skin, muscles, eyes, and lower jaw, fixed in 4% formalin for 1 week and decalcified in Osteosoft (Merck) for 2 weeks. After removing incisors and neurocranium, tissue was embedded in paraffin. Hindpaws were isolated by cutting in the middle of the tibia and removing the skin. The paws were fixed in 4% formalin for 1 week, decalcified in Osteosoft for 2 weeks and embedded in paraffin. LNs were fixed in Carnoy’s solution (ethanol:chloroform:acetic acid 6:3:1) overnight followed by 8 h incubation in 100% ethanol and paraffin embedding. 5 µm paraffin sections were Giemsa-stained for MC quantification. Dermal MCs were counted per 20 mm horizontal length of epidermis. In frontal sections of the snout, MCs were counted in the connective tissue surrounding the NALT per 2 high power fields centered on the right and left NALT and the mean of MC numbers on 15 sections/mouse was calculated. In the hindpaws, MCs were quantified around the tarsal bones per high power field centered on the tarsal bone. MC numbers were quantified for each hind paw of one mouse and the mean was calculated. For cytospins, cells from the peritoneal cavity were isolated by flushing with 5 ml ice cold PBS. Pellets were resuspended in 2% BSA/PBS and 200 µl were loaded per cuvette and spun at 800 rpm for 2 min in a cytocentrifuge (Shandon). Air-dried slides were stained according to standard May-Grünwald and Giemsa protocols. Analysis was performed using Zeiss Axiovision Software.

### Statistical Analysis

Data are shown as mean ± SD unless stated otherwise. For two-group comparisons two-tailed Mann–Whitney test with 95% confidence interval was used. For multi-group comparisons, Kruskal–Wallis with Dunn’s post-test (significance level α = 0.05) was performed. Time-course data were analyzed by two-way ANOVA with Bonferroni post-tests. Chi square test was used to analyze CIA incidence. Significance levels: *p < 0.05, **p < 0.01, and ***p < 0.001.

## Ethics Statement

All procedures were in accordance with institutional guidelines on animal welfare and were approved by the Landesdirektion Dresden (DD24-5131/207/2 and DD24-5131/207/33).

## Author Contributions

NS and KL designed and performed experiments. CA, AW, SG, LM, CH, TH, and LS performed experiments. DV generated *R-DTA* mice. TF and H-RR generated *Cpa3Cre* mice. FG and WM contributed to the design of the study. AD contributed to study design and discussed data. AR conceived and supervised the study. NS and AR wrote the manuscript.

## Conflict of Interest Statement

The authors declare that the research was conducted in the absence of any commercial or financial relationships that could be construed as a potential conflict of interest.
