# Supplementary material for: Unimpaired Responses to Vaccination With Protein Antigen Plus Adjuvant in Mice With Kit-Independent Mast Cell Deficiency
Source: Front Immunol. 2018 Aug 28;9:1870. doi: 10.3389/fimmu.2018.01870 (PMC6123530; doi:10.3389/fimmu.2018.01870)
Supplement: Supplementary file 1 [file data_sheet_1.PDF]

Supplementary Material and Methods

Unimpaired responses to vaccination with protein antigen plus adjuvant in mice with Kit-independent mast cell deficiency

Nadja Schubert[1#], Katharina Lisenko[1#], Christian Auerbach[2], Anke Weitzmann[1], Shanawaz Mohammed Ghouse[1], Lina Muhandes[1], Christa Haase[1], Tobias Häring[1], Livia Schulze[1], David Voehringer[3], Florian Gunzer[2], Werner Müller[4], Thorsten B. Feyerabend[5], Hans-Reimer Rodewald[5], Anne Dudeck[1,6], Axel Roers[1*]

[1] Institute for Immunology, Medical Faculty Carl-Gustav Carus, University of Technology Dresden, Dresden, Germany
[2] Institute of Medical Microbiology and Hygiene, Medical Faculty Carl-Gustav Carus, University of Technology Dresden, Dresden, Germany
[3] Department of Infection Biology, University Hospital Erlangen and Friedrich-Alexander University Erlangen-Nuremberg (FAU), Erlangen, Germany
[4] Faculty of Life Sciences, University of Manchester, Manchester, UK
[5] Division of Cellular Immunology, German Cancer Research Center, Heidelberg, Germany
[6] Institute for Molecular and Clinical Immunology, Medical Faculty, Otto von Guericke University, Magdeburg, Germany
[#] first authors

*** Correspondence:**

Correspondence: Axel Roers
Institute for Immunology
Medical Faculty Carl-Gustav Carus, University of Technology Dresden
Fetscherstraße 74
01307 Dresden, Germany
Phone: ++49 351 458 6500
Fax: ++49 351 458 6316
E-mail: axel.roers@tu-dresden.de

Supplementary Methods

Antibodies. Monoclonal antibodies directed against the following mouse antigens were obtained from eBiosciences: CD11c (N418), CD3 (145-2C11), CD4 (GK1.5), CD8a (53-6.7), CD19 (eBio1D3), CD44 (IM7), CD11b (M1/70), MHC II I-A/I-E (M5/114.15.2), CD117 (2B8), FcεRIα (MAR-1), CD45 (30-F11), CD49b (DX5), F4/80 (BM8), Ly-6C/G (RB6-8C5), NK1.1 (PK136), Siglec-F (E50-2440). Purified antibodies directed against CD3 (17A2) and CD28 (37.51) were used for restimulation of LN cells.

T cell restimulation. To validate the specificity of the tetramer staining for 2W1S-specific cells, $CD44^+$ $tetramer^+$ and $CD44^+$ tetramer-negative cells were sorted using an Aria Cell Sorter III (BD). $2x10^5$ total LN cells from an untreated WT mouse were seeded in 100 µl complete RPMI supplemented with 10 %FCS, 1% Penicillin/Streptomycin, 1% L-alanyl L-glutamine, 1% Sodium pyruvate and 50mM β-Mercaptoethanol per well of a 96-well-plate to provide antigen-presenting cells. 3000 sorted $tetramer^+$ or tetramer-negative T cells were added in 100µl complete RPMI per well. Cells were re-stimulated with anti-CD3 (pre-coated over night at 4°C in PBS) and anti-CD28 (in solution) in a final concentration of 2µg/ml each, or were re-stimulated specifically with 2W1S in a final concentration of 50µg/ml. LN cells plus $tetramer^+$ cells stimulated with irrelevant peptide (MOGp35-55) in a final concentration of 50µg/ml or medium alone, as well as LN cells plus tetramer-negative T cells stimulated with 2W1S peptide served as negative controls. Supernatant was collected after 96h of incubation at 37°C, 5% CO_2 and analyzed for IFN-γ concentration by ELISA (eBioscience).

Quantitative RT-PCR. Total RNA of ear skin, intestine and nasal tissue was isolated using TRIzol® Reagent (ThermoFisher Scientific). Reverse transcription of 1µg total RNA was performed using the RevertAid™ H Minus First Strand cDNA Synthesis Kit (Fermentas) and oligo(dT) primer. 2µl of 1:10 diluted cDNA served as template for quantitative RT-PCR (Maxima® SYBR Green/ROX qPCR Master Mix (Thermo Scientific), 2pmol gene-specific primers) with the following cycling conditions on a Mx3005P QPCR system (Agilent Technologies): 10min 95°C, 40 cycles of 95°C for 30s, 60°C for 30s and 72°C for 25s. Transcript levels were analyzed with the MxPro QPCR Software (Agilent Technologies). The following RT-PCR primers were used: Mcpt1 Fwd 5′-GGA AAA CTG GAG AGA AAG AAC CTA C-3′, Mcpt1 Rev 5′-GAC AGC TGG GGA CAG AAT GGG G-3′, Mcpt5 Fwd 5'-TGC AGT GGC TTC CTG ATA AGA-3‘, Mcpt5 Rev 5'-ACA TAG GAT GCA ATG CCT TGG-3', TBP Fwd 5′-TCT-ACC-GTG-AAT-CTT-GGC-TGT-AAA-3′, TBP Rev 5′-TTC-TCA-TGA-TGA-CTG-CAG-CAA-A-3′. Transcript levels were normalized to TATA box binding protein (TBP). All samples were run in technical triplicates.

Collagen-induced arthritis (CIA). *Mcpt5-Cre* $R26^{iDTR/iDTR}$ and *Mcpt5-Cre* $R26^{DTA/DTA}$ were backcrossed to the CIA-susceptible DBA/1 background for 5 generations. Chicken collagen type II (Chondrex) was dissolved in 0.1M acetic acid over night and emulsified in an equal volume of CFA (with 2mg/ml *Mycobacterium tuberculosis*, Chondrex). Mice were anesthetized with Ketamine/Xylazine and the emulsion was intradermally injected in a total volume of 100µl distributed to both sides of the tail base. Additional Cre-negative controls were injected with sterile 0.9% saline. Joint inflammation was monitored by measurement of hind paw footpad thickness. A clinical score of 0 to 3 was assigned each paw resulting in a maximal score of 12 for one animal [54]. At day 48, mice were sacrificed and hindpaws and skin was sampled. The paws were fixed in 4% formalin for 1 week, decalcified in Osteosoft for 2 weeks and embedded in paraffin. Standard Giemsa, hematoxylin and eosin (H&E) or

Safranin-O staining of 5µm paraffin sections was performed for MC quantification, histological scoring or quantification of cartilage area, respectively. Inflammation was scored on H&E-stained sections (0 - no evidence of inflammation, 1 - mild leukocyte infiltration, 2 - pronounced leukocyte infiltration and mild destruction of cartilage and bone, 3 – dense leukocyte infiltration and pronounced destruction of cartilage and bone).

Supplementary Figures
Figure S1

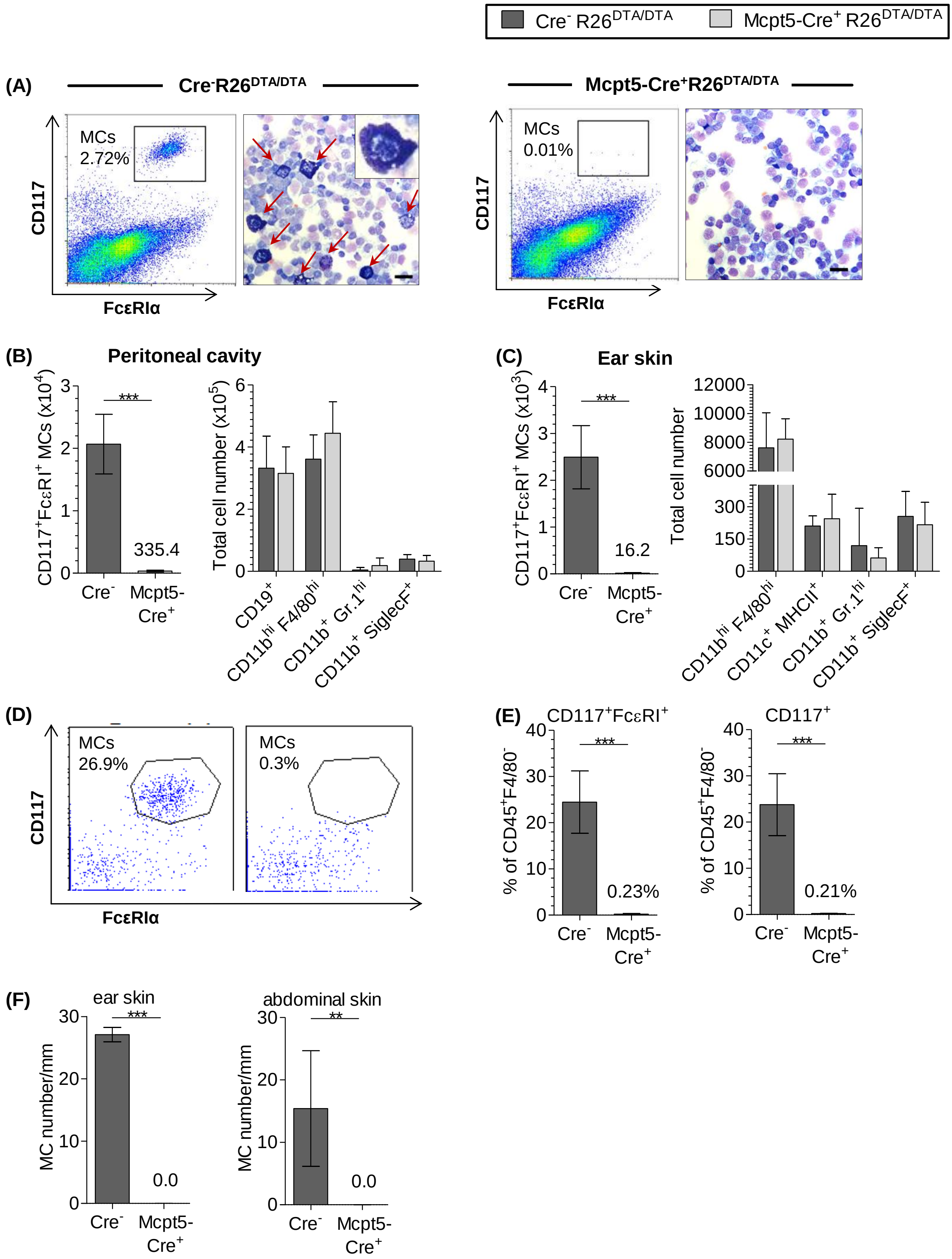

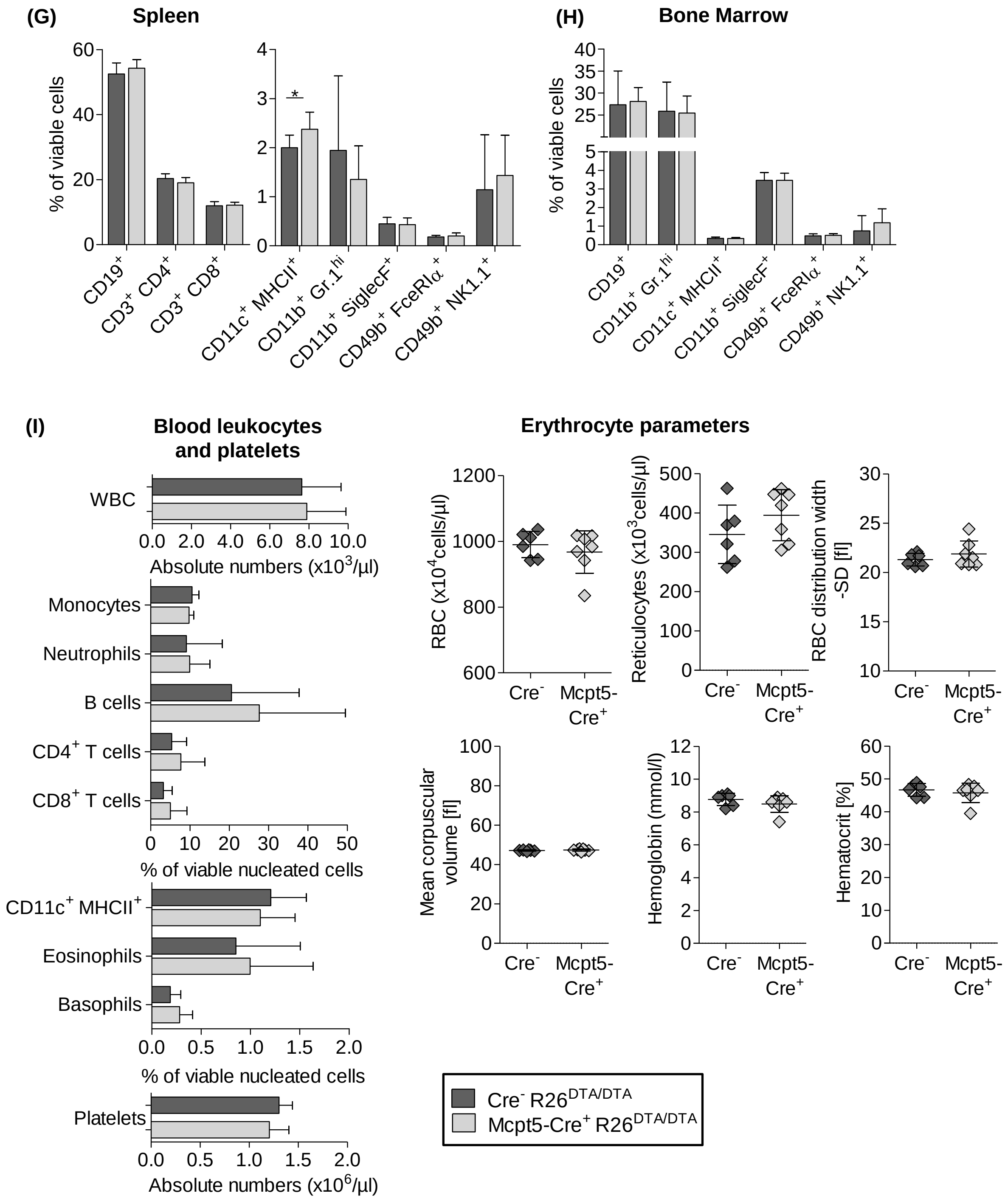

Figure S1. Selective and efficient depletion of CTMCs in *Mcpt5-Cre⁺R26^DTA/DTA* mice.

(A) Flow cytometric analysis and Giemsa-stained cytospins of cells from peritoneal cavity of MC-deficient *Mcpt5-Cre*$^{+}$*R26*$^{DTA/DTA}$ mice (right) and *Cre*-negative littermate controls (left; n=5 per group). Scale bar 20 µm. (B-C) Flow cytometric quantification of various hematopoietic cell types in peritoneal cavity (B) and ear skin (C) of MC-deficient *Mcpt5-Cre*$^{+}$*R26*$^{DTA/DTA}$ mice (n=7) and *Cre*-negative littermate controls (n=7) using immunostaining for the indicated markers. (D) Flow cytometric analysis of MC-deficient *Mcpt5-Cre*$^{+}$*R26*$^{DTA/DTA}$ (right) and *Cre*-negative littermate (left; n=7 per group) ear skin cell suspensions for CD117^{+}FcεRI^{+} expressing cells. Pregated on CD45^{+}F4/80^{-} ear skin cells. (E) Flow cytometric quantification of CD117^{+}FcεRI^{+} MCs (left) or CD117 single positive cells (right) among CD45^{+} F4/80^{-} cells in ear skin suspensions of MC-deficient *Mcpt5-Cre*$^{+}$*R26*$^{DTA/DTA}$ mice and *Cre*-negative

littermate controls (n=7 per group). (F) Quantification of MCs in Giemsa stained sections of Carnoy-fixed ear and abdominal skin of *Mcpt5-Cre*$^{+}$*R26*$^{DTA/DTA}$ (n=6) and Cre-negative littermates (n=4). (G-I) Flow cytometric quantification of various hematopoietic cell types and red blood cell parameters in spleen (G), bone marrow (H) and blood (I) of MC-deficient *Mcpt5-Cre*$^{+}$*R26*$^{DTA/DTA}$ mice (n=7) and *Cre*-negative littermate controls (n=7) using immunostaining for the indicated markers. A Sysmex XT-2000iV Analyzer was used to quantify blood monocytes, platelets and all erythrocyte parameters. Means ± SD are shown. In all cases unpaired two-sided Student’s t-test was used for statistical analysis.

Figure S2

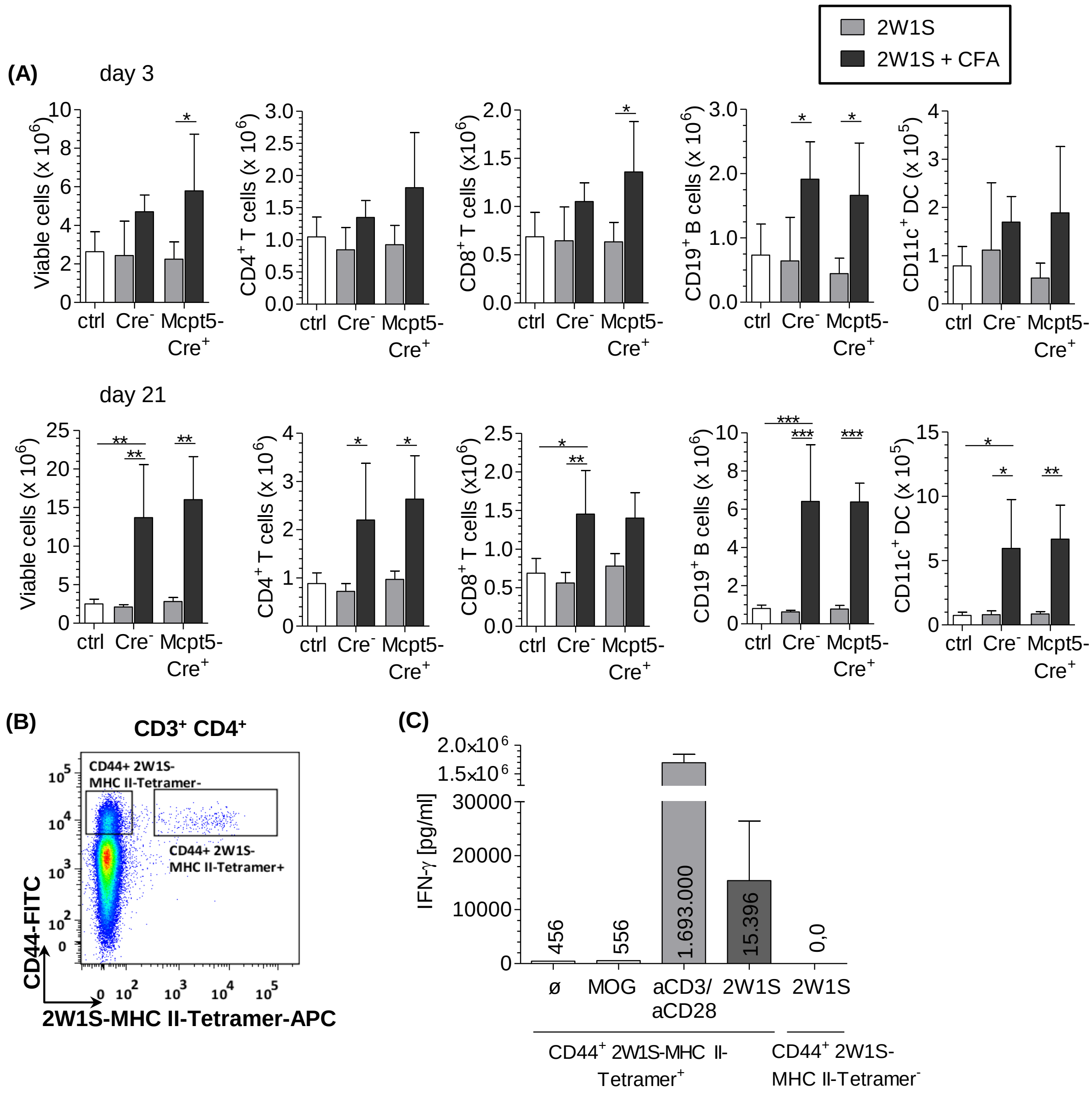

Figure S2. Quantification of LN hypertrophy response upon immunization with 2W1S peptide plus CFA and validation of 2W1S MHCII tetramer staining.

(A) Inguinal LN cellularity analyzed by flow cytometry 3 and 21 days after intradermal immunization of *Cre*-negative *R26*$^{DTA/DTA}$ and *Mcpt5-Cre*$^{+}$*R26*$^{DTA/DTA}$ mice at the tail base with 2W1S peptide plus or without CFA (n=5-7 per group). Saline-treated *Cre*-negative mice served as controls (n=4). Means ± SD are shown. (B) Sorting strategy for the isolation of activated (CD44^{+}) 2W1S-specific (tetramer^{+}) CD3^{+} CD4^{+} T cells of 2W1S/CFA immunized mice. Pregating was on live cells negative for CD11b^{+}, CD11c^{+}, F4/80^{+} and CD19^{+}. (C) *In vitro* restimulation of 3000 2W1S-specific (tetramer^{+}) or tetramer-negative CD4^{+} T cells from C57BL/6 wt mice immunized with 2W1S peptide plus CFA in co-cultures with 2x10^{5} total LN cells from untreated wt mice. IFN-γ concentrations in the supernatant 96 h after restimulation with 2W1S peptide, an irrelevant control peptide (myelin oligodendrocyte glycoprotein, MOG) or anti-CD3/anti-CD28 were determined by ELISA. In all cases statistical analysis was performed using one-way ANOVA and Bonferroni's multiple comparison test.

Figure S3

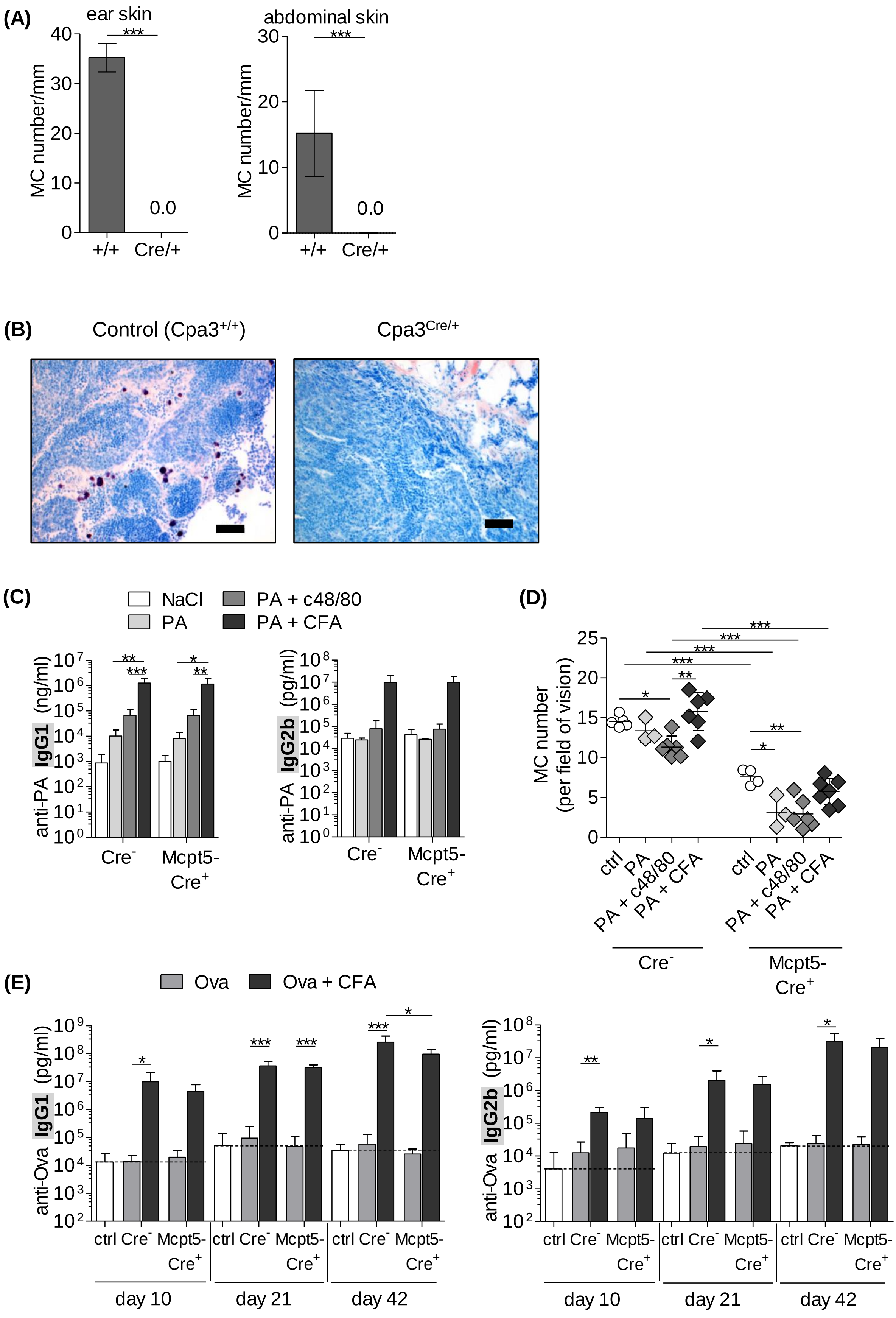

(A)
ear skin
abdominal skin
MC number/mm
***
0.0
+/+
Cre/+
(B)
Control (Cpa3+/+)
Cpa3Cre/+
(C)
NaCl
PA
PA + c48/80
PA + CFA
anti-PA IgG1 (ng/ml)
anti-PA IgG2b (pg/ml)
Cre-
Mcpt5-Cre+
(D)
MC number (per field of vision)
ctrl
PA
PA + c48/80
PA + CFA
Cre-
Mcpt5-Cre+
(E)
Ova
Ova + CFA
anti-Ova IgG1 (pg/ml)
anti-Ova IgG2b (pg/ml)
ctrl
Cre-
Mcpt5-Cre+
day 10
day 21
day 42

Figure S3. The antibody response to immunization with protein antigen is not impaired by reduced MC numbers or MC-specific knock out of TNF.

(A) Quantification of MCs in Giemsa stained sections of Carnoy-fixed ear and abdominal skin of untreated *Cpa3*$^{Cre/+}$ and *Cpa3*$^{+/+}$ mice (both groups n=4).

(B) To verify MC deficiency in the draining LNs of *Cpa3*$^{Cre/+}$ mice and *Cpa3*$^{+/+}$ littermates, we fixed inguinal LNs in Carnoy's solution at day 42 after intradermal immunization with Ova plus CFA and prepared Giemsa-stained paraffin sections. Representative histologies (scale bar 50 µm) of LNs from MC-deficient *Cpa3*$^{Cre/+}$ mice and *Cpa3*$^{+/+}$ littermates are shown.

(C) *Mcpt5-Cre*$^{+}$*R26*$^{DTA/wt}$ mice and *Cre*-negative littermate controls were immunized with *B. anthracis* protective antigen (PA) alone (n=3 both groups), PA plus c48/80 (n=6 both groups) or PA plus CFA (*Cre*$^{+}$ n=7, *Cre*$^{-}$ n=6). Serum concentrations of PA-specific IgG1 and IgG2b were determined 21 days later. Saline (NaCl)-treated animals (n=9, 5 *Cre*$^{-}$ and 4 *Cre*$^{+}$) served as negative controls.

(D) Quantification of MCs in the skin tissue of the immunization site sampled from the animals represented in (A) at day 21. MCs were counted in Giemsa-stained sections in a total of 20 randomly placed fields of vision per animal. Mean ± SD are shown

(E) *Mcpt5-Cre*$^{+}$*TNF*$^{FL/FL}$ mice and *Cre*-negative littermate controls were intradermally immunized with Ova alone, Ova plus c48/80 or Ova plus CFA (n=5 all groups) and serum concentrations of Ova-specific IgG1 and IgG2b were determined 10, 21 and 42 days later. Saline treated *Cre*-negative mice (n=7) served as controls. Means ± SD are shown. Statistical analysis was performed using unpaired two-sided Student's t-test (A) or one-way ANOVA and Bonferroni's multiple comparison test (C-E).

Figure S4

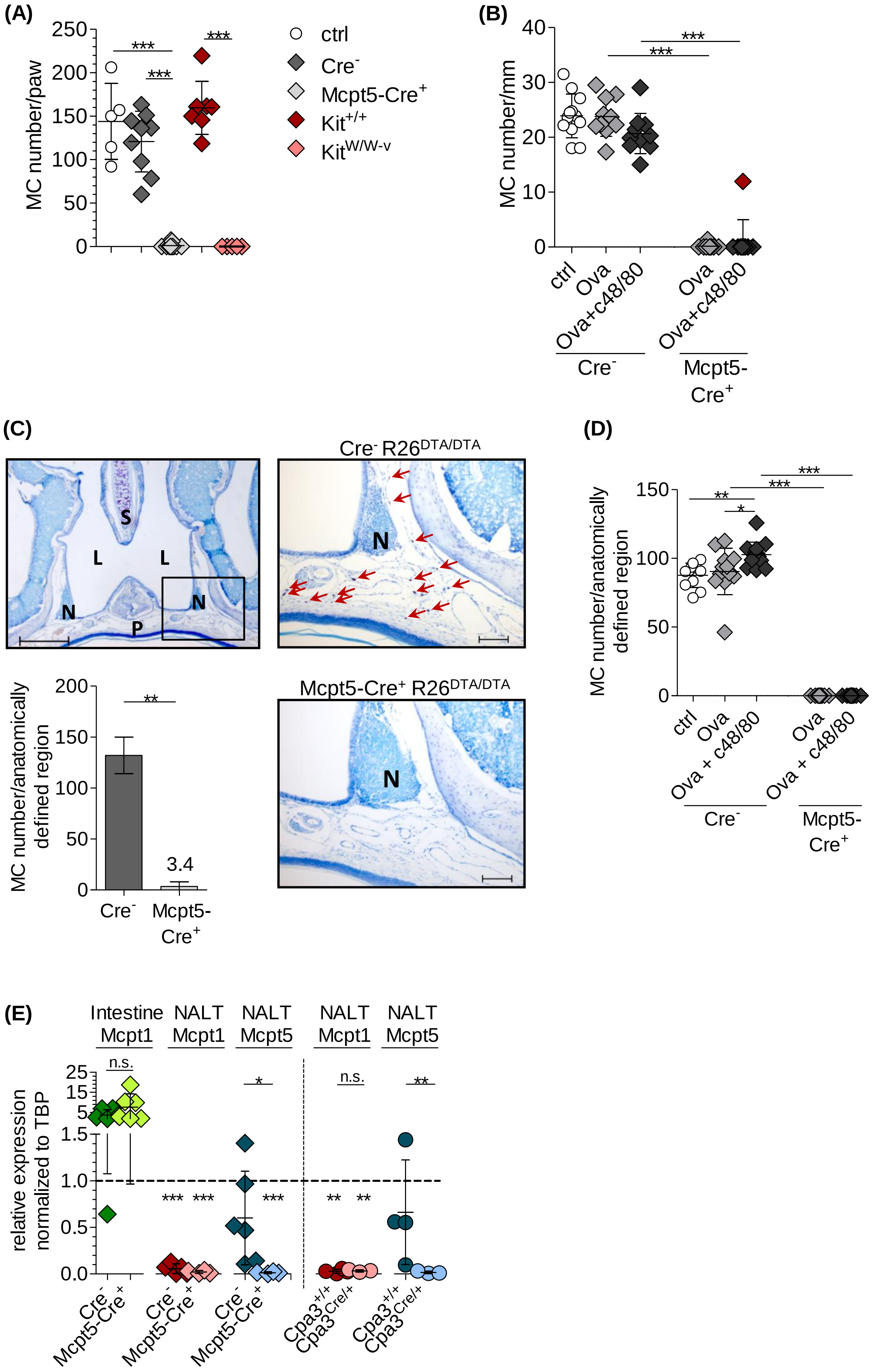

(A)
MC number/paw
ctrl
Cre-
Mcpt5-Cre+
Kit+/+
KitW/W-v
(B)
MC number/mm
ctrl
Ova
Ova+c48/80
Cre-
Mcpt5-Cre+
(C)
S
L
N
P
Cre- R26DTA/DTA
Mcpt5-Cre+ R26DTA/DTA
MC number/anatomically defined region
3.4
(D)
MC number/anatomically defined region
ctrl
Ova
Ova + c48/80
Cre-
Mcpt5-Cre+
(E)
Intestine Mcpt1
NALT Mcpt1
NALT Mcpt5
NALT Mcpt1
NALT Mcpt5
relative expression normalized to TBP
n.s.
Cre-
Mcpt5-Cre+
Cpa3+/+
Cpa3Cre/+

Figure S4. Efficiency of MC depletion at dermal and mucosal immunization sites.

(A) MC numbers in hindpaw tissue sampled from all animals in the experiment represented in Fig. 4A 24 h after injection of c48/80 or saline into the footpads or (B) in tail base skin tissue sampled from all animals in the experiment represented in Fig. 4C at day 42 after immunization. MCs were counted in Giemsa-stained tissue sections per foot or 20 mm length of epidermis for each mouse. In (B), one *Mcpt5-Cre*$^{+}$ mouse (shown in red) was incompletely MC depleted and was excluded from the analysis. (C) Representative histologies (scale bars: left 500 µm, right 100 µm) and quantification of MCs in Giemsa-stained frontal sections of decalcified facial skull from untreated *Mcpt5-Cre*$^{+}$*R26*$^{DTA/DTA}$ and *Cre*-negative littermates (n=7 both groups). For each animal, MCs were counted in the connective tissue surrounding the NALT per 2 high power fields centered on the left and right NALT in at least 15 sections containing both (left and right) NALT regions.

(D) MC numbers in nasal tissue sampled from all animals in the experiment represented in Fig. 4E 42 days after mucosal immunization.

(E) Nasal mucosa of control mice does not contain Mcpt1 mRNA expressing mucosal mast cells and Mcpt5 mRNA expressing CTMCs are efficiently depleted in *Mcpt5-Cre*$^{+}$*R26*$^{DTA/DTA}$ and *Cpa3*$^{Cre/+}$ nasal tissue. Relative transcript levels of Mcpt1 (transcribed in MMCs) and Mcpt5 (transcribed in CTMCs) in the intestine (positive control) and nasal tissue of untreated *Mcpt5-Cre*$^{+}$*R26*$^{DTA/DTA}$ and *Cpa3*$^{Cre/+}$ mice and the respective *Cre*-negative controls normalized to TATA-binding protein (TBP) transcript levels, which were set to 1 (n=4-6 per group). Mcpt1 transcript levels were very low or undetectable in the nasal mucosa from animals of all genotypes but readily detected in intestinal tissue. Mcpt5 mRNA was detected in control mice but not in *Mcpt5-Cre*$^{+}$*R26*$^{DTA/DTA}$ and *Cpa3*$^{Cre/+}$ mice. Means ± SD are shown.

Statistical analysis was performed using one-way ANOVA and Bonferroni's multiple comparison test (A, B, D,E) or unpaired two-sided Student's t-test (C).

Figure S5

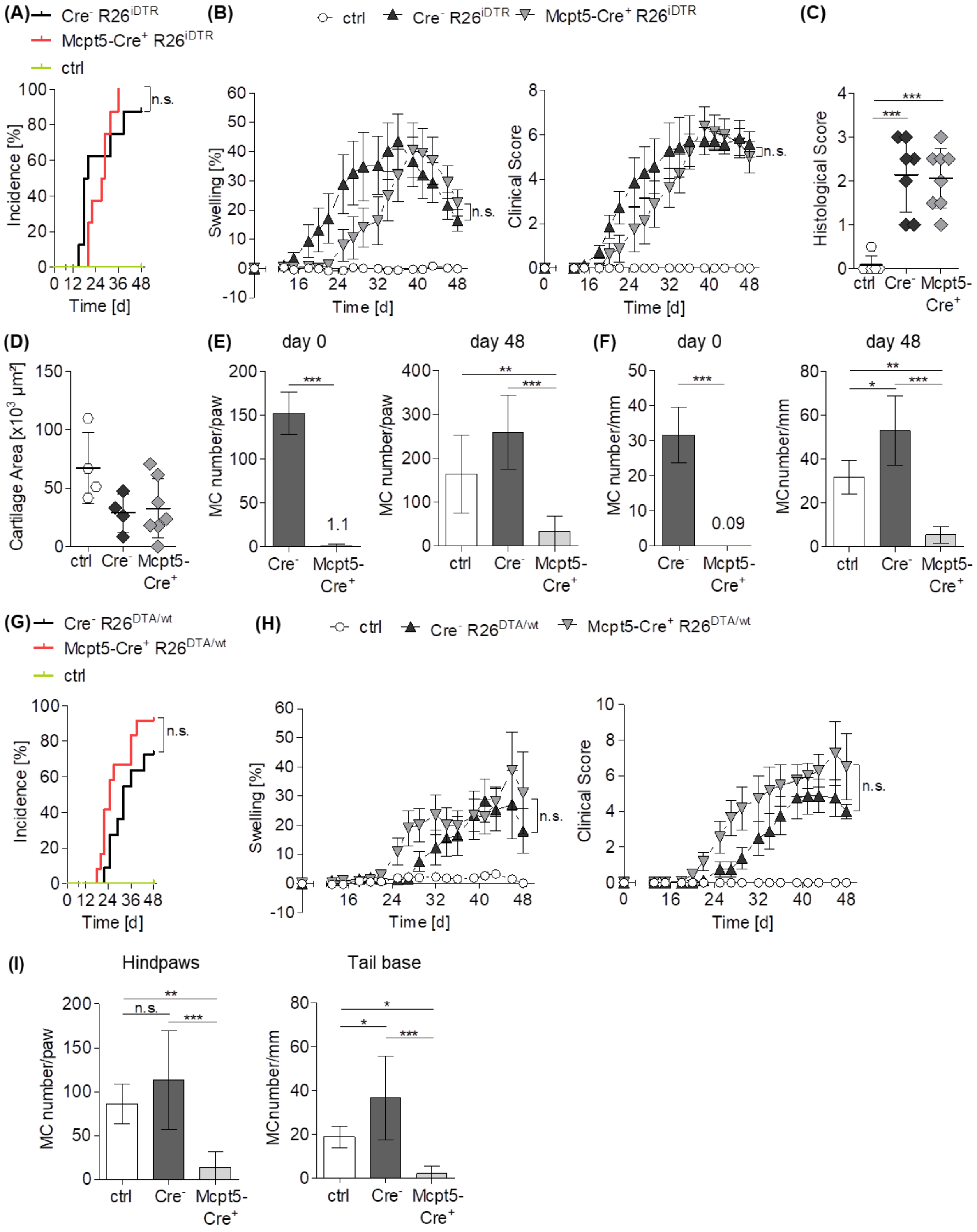

Figure S5. Collagen-induced arthritis (CIA) is not diminished in MC-deficient DBA/1 mice.

CIA was induced in *Mcpt5-Cre R26$^{iDTR/wt}$* mice in which MC-deficiency was induced by injection of diphtheria toxin [30] (A-E) and in constitutively MC-deficient *Mcpt5-Cre R26$^{DTA/wt}$* (F-G). Both models were back-crossed onto the CIA-susceptible background DBA/1 for 5 generations.

(A) Incidence, (B) footpad swelling of hindpaws and clinical score of *Mcpt5-Cre R26$^{iDTR/wt}$* mice ('Mcpt5-Cre^{+}', n=8) and their Cre-negative littermate controls ('Cre^{-}', n=8) responding to immunization with Collagen II/CFA and additional DT-treated Cre-negative controls injected with saline ('ctrl', n=6). All animals had been injected i.p. with 25ng/g body weight DT at weekly intervals for four weeks, with the last DT injection 4 days before the experiment. Data represent means ± SEM. Statistical analysis was performed using log-rank test (A) or two-way ANOVA with Bonferroni's post-tests (B). (C) Histological score and (D) cartilage area were determined in paraffin embedded hindpaws from the mice represented in Fig. S5A and B, stained with H&E or Safranin-O, respectively. Data represent means ± SD. One-way ANOVA and Bonferroni's multiple comparison test was used for statistical analysis.

(E, F) Efficient MC depletion in hindpaw tissue (E) and at the immunization site at the tail base (F) was verified in Giemsa-stained skin sections of untreated mice (day 0) and at day 48 after immunization (*Cre^{-}* n=11, *Cre^{+}* n=9 at day 0; *Cre^{-}* n=7, *Cre^{+}* n=8, ctrl n=6 at day 48). Data represent means ± SD. Statistical analysis was performed using unpaired two-sided Student's t-test (day 0) or one-way ANOVA and Bonferroni's multiple comparison test (day 48).

(G) Incidence, (H) footpad swelling of the hindpaws and clinical score of *Mcpt5-Cre R26$^{DTA/wt}$* mice responding to immunization with Collagen II/CFA (*Cre^{-}* n=11, *Cre^{+}* n=12). Additional Cre-negative animals injected with saline instead of collagen/CFA (n=5) served as negative controls. Means ± SEM are shown. Statistical analysis was performed using log-rank test (G) or two-way ANOVA with Bonferroni's post-tests (H).

(I) MC numbers in hindpaw tissue and at the immunization site at the tail base in Giemsa-stained sections of tissue sampled at day 48 after immunization (*Cre^{-}* n=10, *Cre^{+}* n=11, ctrl n=5). Data represent means ± SD. One-way ANOVA and Bonferroni's multiple comparison test was used for statistical analysis.
